# The effects of interleukin-1β in modulating osteoclast-conditioned medium's influence on gelatinases in chondrocytes through mitogen-activated protein kinases

**DOI:** 10.1038/ijos.2015.39

**Published:** 2015-10-30

**Authors:** Jing Xie, Na Fu, Lin-Yi Cai, Tao Gong, Guo Li, Qiang Peng, Xiao-Xiao Cai

**Affiliations:** State Key Laboratory of Oral Diseases, West China Hospital of Stomatology, Sichuan University, Chengdu, China

**Keywords:** chondrocyte, gelatinases, interleukin-1β, matrix crosstalk, osteoarthritis, osteoclast

## Abstract

Osteoarthritis is recognised to be an interactive pathological process involving the cartilage, subchondral bone and synovium. The signals from the synovium play an important role in cartilage metabolism, but little is known regarding the influence of the signalling from bone. Additionally, the collagenases and stromelysin-1 are involved in cartilage catabolism through mitogen-activated protein kinase (MAPK) signalling, but the role of the gelatinases has not been elucidated. Here, we studied the influence of osteoclastic signals on chondrocytes by characterising the expression of interleukin-1β (IL-1β)-induced gelatinases through MAPK signalling. We found that osteoclast-conditioned media attenuated the gelatinase activity in chondrocytes. However, IL-1β induced increased levels of gelatinase activity in the conditioned media group relative to the mono-cultured chondrocyte group. More specifically, IL-1β restored high levels of gelatinase activity in c-Jun N-terminal kinase inhibitor-pretreated chondrocytes in the conditioned media group and led to lower levels of gelatinase activity in extracellular signal-regulated kinase or p38 inhibitor-pretreated chondrocytes. Gene expression generally correlated with protein expression. Taken together, these results show for the first time that signals from osteoclasts can influence gelatinase activity in chondrocytes. Furthermore, these data show that IL-1β restores gelatinase activity through MAPK inhibitors; this information can help to increase the understanding of the gelatinase modulation in articular cartilage.

## Introduction

Osteoarthritis (OA), a chronic and degenerative joint disease, has the highest prevalence among the arthritic maladies.^1^ Although cartilage degradation is its main feature, the disease is now considered to be an irreversibly destructive cascade of the entire joint involving the synovium, subchondral bone and cartilage.^2^ During the pathological process, different cell types from these tissues interact and influence each other as an integrated network.^3^ Previous studies have identified that chondrocytes and synovial cells can communicate either by direct contact^4^ or indirectly by synovial cell release of molecular agents.^5^ It has also been demonstrated that macrophages from the synovium can influence the chondrocytes.^6^ However, little is known about the potential soluble factors from osteoclasts in the subchondral bone to the influence on chondrocytes.

Several cytokines are known to be potent mediators of cartilage metabolism.^7^ Of these, interleukin-1β (IL-1β) is one of the most important factors in OA, and it can potently induce cartilage catabolic agents and suppress proteoglycan and collagen synthesis.^8^ It is known that an enhanced catabolism disrupts the balance between matrix synthesis and degradation in the pathogenesis and progression of OA.^9,10^ The metalloproteinases (MMPs), which are promoted by IL-1β, contribute to this process.^7^ The collagenases (MMP-1, -8 and -13) play a vital role in the irreversible breakdown of cartilage matrix via the direct digestion of type II collagen and the consequent release of matrix proteoglycan from the cartilage.^11^ Moreover, they can denature the fibrillar collagens (*i.e.*, type I, II and III).^9^ The stromelysins (MMP-3, -10 and -11) are able to degrade the collagens (*e.g.*, type II, III, IX and XI) and proteoglycans and participate in the activation of other pro-MMPs (*e.g.*, pro-collagenases).^10^ The gelatinases (gelatinase A, MMP-2, 68 kDa type IV collagenase and gelatinase B, MMP-9, 87 kDa type IV collagenase), which are the second step for the extracellular matrix (ECM) degradation followed by the direct collagenases, can further degrade the collagenase-denatured collagens (gelatin). The gelatinases can also activate other MMPs, such as the collagenases.^12^

IL-1β-induced MMPs are mainly involved in the activation of the mitogen-activated protein kinase (MAPK) family, which relays extracellular stimuli into intracellular signals.^13^ The MAPK family can be divided into three major cytoplasmic pathways: the extracellular signal-regulated kinase (ERK1/2), p38 and c-Jun N-terminal kinase (JNK). These three MAPK conduits rank among the most extensively studied signal transduction networks and have been demonstrated to contribute to a diverse function in cells.^14^ In articular chondrocytes, previous data have shown that IL-1β-mediated MMP-1 (collagenase-1) expression requires the activation of ERK and/or p38.^15^ Thus, the specific inhibitors of ERK (PD98059) and/or p38 (SB203580) can diminish the effect of IL-1β on MMP-1 expression.^15^ IL-1β-enhanced MMP-13 (collagenase-3) expression not only requires the activation of p38 kinase ^16^ but also relies on JNK activity.^17^ Thus, inhibition of JNK with SP600125 can inhibit MMP-13 expression.1^6,18^ As the main stromelysin expressed in chondrocytes, MMP-3 (stromelysin-1) is upregulated by IL-1β through the activation of the MAPKs.^16,19^ Again, the specific inhibitors of the MAPKs can also reduce the expression of MMP-3.^20^ The gelatinases, which are predominantly expressed in the deep zone of cartilage, play critical roles in the homeostatic balance of ECM in the hinge region between the cartilage and bone.^21^ However, the regulatory mechanisms of IL-1β-induced gelatinases in articular chondrocytes involving MAPK signalling pathway are not well understood.

Spontaneous OA animal models and patients show that increased bone resorption occurs at an early stage in the development of OA, and blocking bone-resorbing factors prevents cartilage damage.^22,23^ Based on this evidence, we hypothesised the following: (1) the gelatinases produced by chondrocytes could be modulated through secreted factors from osteoclasts; and (2) IL-1β-induced gelatinases are regulated by MAPK signalling pathways. To test our hypotheses, we collected conditioned media from osteoclasts to study how the activity of gelatinases in chondrocytes was influenced by secreted factors from osteoclasts. IL-1β was then added to the media to induced gelatinase expression. Finally, we examined the gene expression of the gelatinases and tissue inhibitors of metalloproteinases (TIMPs) in chondrocytes treated with or without MAPK inhibitors.

## Methods and materials

### Chondrocyte culture

The animal materials used for this study were obtained according to ethical principles and the protocol was reviewed and approved by our Institutional Review Board. Chondrocytes were isolated from the knee joint of a 1-day-old mouse. Briefly, the mouse was killed and sterilised, and then the knee joint was collected with ophthalmic scissors. The epidermis of the knee joint was stripped. The collected knee joint was cut into small pieces and trypsinised (0.25%) for 30 min. The trypsin-contained supernatant was then removed and replaced with 0.5% collagenase type II for 3 h. The collagenase type II-treated solution (chondrocyte suspension) was collected and mixed 1:1 (*V*/*V*) with fresh 10% heat-activated foetal bovine serum (FBS), Dulbecco's modified Eagle's medium (DMEM) (high-glucose DMEM, 0.1 mmol·L^−1^ non-essential amino acids, 4 mmol·L^−1^ L-glutamine, 1% penicillin–streptomycin solution). The mixed suspension was centrifuged at 1 000 r·min^−1^ for 5 min. After removing the supernatant, the 10% FBS DMEM was added into centrifuge tube to resuspend the chondrocytes. Then, the suspended chondrocytes were seeded into plates or flasks at 37 °C in a humidified atmosphere of 5% CO_2_ till usage.

### Collection of osteoclast conditioned media

Multinucleated osteoclasts (mouse source) were induced as previously described.^24^ Briefly, we adopted the conditioned media from MC3T3-E1 osteoblasts to induce the fusion of RAW264.7 monocytes, through which multinucleate osteoclasts have been demonstrated to form. RAW264.7 cells were seeded at a density of 2 × 10^4^ cells per cm^2^ on plastic coverslips. After 24 h, the media were replaced with a 1:1 (*V*/*V*) mixture of the conditioned media and the growth media of DMEM supplemented with 10% heat-inactivated FBS, 3.5 mmol·L^−1^ L-glutamine and 1% penicillin–streptomycin solution. The time point prior to the induction was described as ‘0 day'. The induction media containing the conditioned medium was changed every other day. The multinucleated osteoclasts with more than two nuclei were visible after 2 days induction. The induction maintained and equilibrated for next 24 h, and then we reduced FBS concentration to 2% for 16 h starvation. After starvation, the media were changed to fresh 1% FBS DMEM. The osteoclast-conditioned media were collected in 1% FBS DMEM for 72 h.

Chondrocytes were seeded onto six-well plates with 10% heat-activated FBS DMEM. After equilibration for 24 h, the culture medium was replaced with 2% FBS DMEM for a 16 h starvation. Then, the chondrocyte culture medium was replaced with a 1:1 (*V*/*V*) mixed media of collected conditioned medium and 1% FBS DMEM. At 12, 24, 48 and 72 h after incubation, 300 μL of media samples were collected for zymography. Cell lysate samples (1 000 µL) were collected at 2 h for semi-quantitative polymerase chain reaction (PCR).

### IL-1β treatment

Chondrocytes were seeded onto six-well plates at 5 × 10^5^ cells per well (85%–95% confluence). Chondrocytes were allowed to equilibrate for 24 h. The culture media were then replaced with 2% FBS DMEM for a 16 h starvation. Then, chondrocytes were split into two groups. In the mono-culture group, the media were replaced with fresh 1% FBS DMEM containing different concentrations (1, 5, 10 and 20 ng·mL^−1^) of IL-1β (Peprotech, Rocky Hill, NJ, USA). In the co-culture group, IL-1β was added into the 1:1 (*V*/*V*)-mixed media (osteoclast-conditioned media and 1% FBS DMEM) for chondrocytes culture. Media samples (300 μL) were collected at 12, 24, 48 and 72 h for zymography. At the gene level, 1 000 μL cell lysate samples were collected at 2 h in the mono-culture and co-culture group after treatments with IL-1β at different concentrations; these lysates were used for semi-quantitative PCR.

### Inhibitor treatment

The pretreatments of chondrocytes before starvation were the same as described above. After a 16-h starvation, the culture medium was replaced with fresh 1% FBS DMEM in the mono-culture group or into the 1:1 (*V*/*V*) mixed media (osteoclast-conditioned media and 1% FBS DMEM) in the co-cultured group. The specific inhibitors of MAPKs (ERK: PD98059 50 μmol·L^−1^, p38: SB203580 20 μmol·L^−1^ and JNK: SP600125 20 μmol·L^−1^, purchased from Sigma, St. Louis, MO, USA) and nuclear factor-κB (NF-κB; Bay11-7082, 20 μmol·L^−1^, from Sigma, St. Louis, MO, USA) were then immediately added into the mono-culture and co-culture groups, respectively. Media samples (300 μL) were collected at 12, 24, 48 and 72 h for zymography. Samples of 1 000 μL cell lysate were collected at 2 h for semi-quantitative PCR. To study the role of IL-1β in inhibitor-pretreated chondrocytes, IL-1β was added into the mono-culture and co-culture chondrocytes after 30 min of incubation with inhibitor.

### Cell viability assay

The chondrocytes that were seeded on the six-well plates with different concentrations of IL-1β and MAPKs and NF-κB inhibitors were trypsinised and added to the 96-well plate (Corning, Corning, NY, USA) with resazurin at the concentration of 90 μg·mL^−1^ to 150 μg·mL^−1^ (according to the manufacturer's protocol; TOX8-1KT; Sigma, St. Louis, MO, USA). Resazurin solutions (10 μL) were added into 100 μL cell suspensions for sample detection, and 10 μL of resazurin added into 100 μL of fresh culture media served as the control. The media mixtures were incubated for 2 h at room temperature (RT) before detecting the absorbance at 570 and 600 nm using a BioTek ELx800 (BioTek, Winooski, VT, USA). The samples included both the control group and IL-1β- and inhibitor-treated groups; all treated groups were normalised to the control group.

### Semi-quantitative PCR

Chondrocyte RNA samples were isolated using the RNeasy Plus Mini Kit (Qiagen, Valencia, CA, USA) with a genomic DNA eliminator. Isolated RNA was dissolved in RNase-free water and quantified by measuring the absorbance at 260 nm with a spectrophotometer. The RNA samples were then treated with DNase I (Thermo Fisher Scientific, Waltham, MA, USA), and cDNA was prepared from each sample, using 0.5 μg of total RNA and the cDNA synthesis kit (Thermo Fisher Scientific, Waltham, MA, USA) in a final volume of 20 µL.

To evaluate the expression levels of gelatinases in different treated groups as normalised to the glyceraldehyde-3-phosphate dehydrogenase (GAPDH) and β-actin, semi-quantitative PCR was performed with a PCR kit (Thermo Fisher Scientific, Waltham, MA, USA), using a thermo-cycler (Bio-Rad, Hercules, CA, USA). The selected sets of primers are shown in Table 1. Basic local alignment search tool (BLAST) was used to search for all primer sequences to ensure gene specificity. Semi-quantitative PCRs were performed in a 25 µL volume containing a 1 µL cDNA sample. The PCR programme consisted of a 30-s denaturisation cycle at 94 °C, a 30 s annealing cycle at 55–65 and 72 °C, a 30 s elongation cycle, 25–28 amplification cycles. The products were resolved by 2% agarose gel electrophoresis in trisborate/ethylenediaminetetraacetic acid buffer and visualised by staining with ethidium bromide.

### Quantitative real-time PCR

Quantitative real-time PCR (qPCR) was performed with a QuantiTect SYBR Green PCR Kit (Qiagen, Frankfurt, German) using iCycler (Bio-Rad, Hercules, CA, USA) according to operation procedure. qPCR reactions were performed at 0.5 µmol·L^−1^ for each primer in a 25 µL volume containing 1 µL cDNA sample. The reaction was initiated by activating the polymerase with a 5-min pre-incubation at 95 °C. Amplification was achieved with 45 cycles of 15 s denaturation at 94 °C, 15 s annealing at 64 °C and 15 s elongation at 72 °C. The programme was concluded by the melting curve analysis. All experiments were performed in triplicates. The copy numbers of each gene were determined by cycle threshold (*Δ*Ct) methods. Means of the copy numbers of GAPDH were used as internal controls to normalise the data. The standards for establishing standard curves of all primers were prepared from total normal RNA, amplified by qPCR and cloned by TOPO II TA cloning kit (Invitrogen, Carlsbad, CA, USA), according to the manufacturer's recommendations.

### Zymography

The activities chondrocyte-secreted MMP-2 and -9 were assayed from culture media samples using 0.05% gelatin zymography. Briefly, the protein concentrations were determined using the BCA kit (Kegen, Nanjing, China); the same quantities of different samples were mixed with an equal amount of laemmli sample buffer (62.5 mmol·L^−1^ tris(hydroxymethyl)aminomethane (Tris)-HCl, pH 6.8, 25% glycerol, 2% sodium dodecyl sulphate (SDS), 0.01% bromophenol blue, no β-mercaptoethanol) and separated on a 10% SDS-polyacrylamide gel electrophoresis (PAGE) gel that was co-polymerised with 0.05% gelatin. To regain enzyme activity by removing the SDS, gels were washed three times for 1.5 h in 2.5% Triton X-100 at RT after electrophoresis. Washed gels were then bathed in proteolysis buffer (50 mmol·L^−1^ CaCl_2_, 0.5 mol·L^−1^ NaCl, 50 mmol·L^−1^ Tris, pH 7.8) and incubated at 37 °C for 12–16 h. Following this incubation, the gels were rinsed in a 2.5% Triton X-100 solution and stained at RT with Coomassie blue (45% methanol, 44.75% H_2_O, 10% acetic acid, 0.25% Coomassie blue R-250) for 1 h on a rotator. De-staining was performed (40% methanol, 7.5% acetic acid, 52.5% H_2_O) until white bands appeared clearly from the Coomassie blue background. Bands were scanned using a densitometer (Bio-Rad, Hercules, CA, USA) and quantified using Quantity One 4.6.3 software (Bio-Rad, Hercules, CA, USA). In addition, as the 87 kDa pro-MMP-9 and 68 kDa pro-MMP-2 had only approximately 10% of the activity of the 83 kDa MMP-9 and 65 kDa active-MMP-2, respectively, the bands for the 83 kDa active form MMP-9 and 65 kDa active form MMP-2 was calculated at 10 times the density of the 87 kDa pro-MMP-9 and 68 kDa pro-MMP-2 bands as described previously.^25^

### Enzyme-linked immunosorbent assay

The culture media from monolayer and cultured chondrocytes were collected and the protein amounts of each component in the culture medium were determined. We used the enzyme linked immunosorbent assay (ELISA) kit from Abcam (MMP-2 Mouse ELISA Kit, ab100730 and MMP-9 Mouse ELISA Kit, ab100732; Cambridge, UK). The quantitative measurements were strictly followed by the protocols provided by the manufacturer. Quadruplicate assays were performed on each sample and the absorbance at 450 nm was recorded. Additionally, before the sample loading the culture media were diluted 10-folds in sample preparation.

### Statistical analysis

Statistical analysis was performed by one-way analysis of variance to determine whether differences existed among groups. Post-hoc analysis utilised Fisher's protected least significant differences. In each analysis, the critical significance level was set to be *P* < 0.05.

## Results

### Conditioned media of osteoclasts attenuate the activity of gelatinases secreted by chondrocytes

We first examined the viability of chondrocytes in response to IL-1β (1–20 ng·mL^−1^), PD98059 (ERK inhibitor, 20–60 μmol·L^−1^), SB203580 (p38 inhibitor, 10–30 μmol·L^−1^), SP600125 (JNK inhibitor, 10–30 μmol·L^−1^) and Bay11-7082 (NF-κB, 5–30 μmol·L^−1^). The cell viabilities showed no differences between the control group and treated groups. The concentration ranges were reported in our previous data.^25^

**Figure 1:**
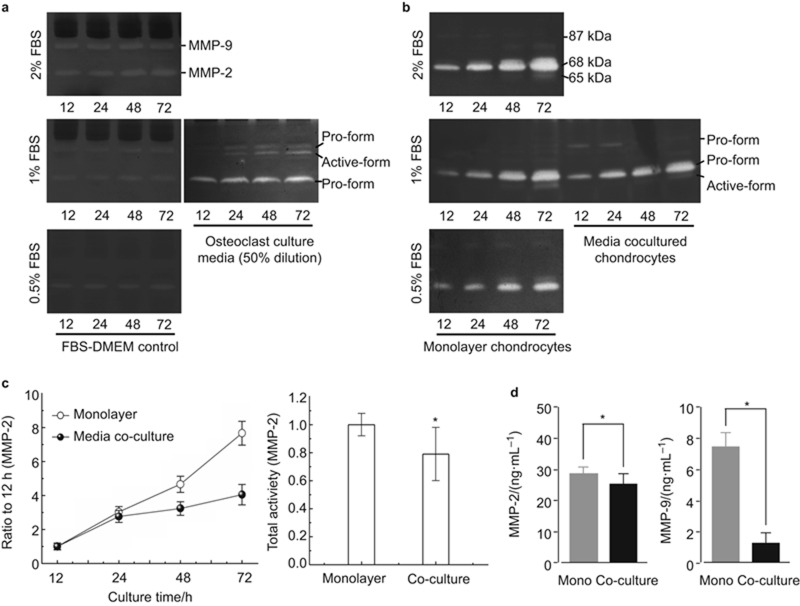
**Osteoclast-conditioned media attenuate the activity of gelatinases secreted by chondrocytes.** (**a**) Zymography demonstrating gelatinases in 0.5%, 1% and 2% fresh FBS culture media and collected osteoclast culture media. Osteoclast culture media (50%) shows the samples of this group loaded for electrophoresis were 50% dilution compared with other groups. (**b**) Zymography demonstrating the activity of gelatinases secreted by mono-cultured and co-cultured chondrocytes. 0.5%, 1% and 2% FBS show the active gelatinase contents in chondrocytes after culture with 0.5%, 1% and 2% FBS; monolayer chondrocytes and media co-cultured chondrocytes are shown in a gel to make a comparison. The gels are the representative of three different experiments (*n* = 3). (**c**) Quantification demonstrated time-dependent increases of MMP-2 in both mono-culture and co-culture chondrocytes. Quantification was performed with Quantity One 4.6.3 software. The optical densities of the pro- and active-MMP-2 bands were added as the total value of activity for MMP-2. The values at 24, 48 and 72 h were compared to the values at 12 h. The quantitative data about total activity refer to 72 h time points of the mono-cultured and co-cultured chondrocytes. The data are the mean of three different experiments (*n* = 3). *Significant difference with respect to monolayer chondrocytes (*P* < 0.05). (**d**) ELISA Kit confirmed the gelatinases secreted by chondrocytes in mono-culture and co-culture groups (mean ± standard deviation) (*n* = 4). *Significant difference with respect to monolayer chondrocytes (*P* < 0.05). DMEM, Dulbecco's modified Eagle's medium; ELISA, enzyme linked immunosorbent assay; FBS, foetal bovine serum; Mono, mono-culture.

We then studied the influence of secreted factors from osteoclasts on chondrocytes by culturing chondrocytes with osteoclast-conditioned media. The activity of the ECM-degrading enzyme gelatinases was measured (Figure 1). After conditioned medium co-culture, we found that the MMP-2 secreted by chondrocytes was reduced compared to secretion from mono-culture chondrocytes. Moreover, the active form (65 kDa) of MMP-2 was only present at very low levels (Figure 1b). Time-accumulated quantification of MMP-2 production demonstrated that MMP-2 activity was lower in the co-culture group than the mono-culture group (Figure 1c). At 72 h after co-culture, total MMP-2 activity was reduced to 79% of the activity in the mono-culture group. In contrast, normal chondrocytes had low levels of MMP-9 secretion (left lane in Figure 1b), while osteoclasts expressed significant levels of pro- and active-MMP-9 (right lane in Figure 1a, the culture media samples from osteoclasts were diluted to 50% for zymography compared with other groups). After co-culture, MMP-9 from osteoclasts was detected in the media at 12 and 24 h (bottom right lane in Figure 1b), but the expression was reduced after 48 h. ELISA also confirmed the significant reduction of MMP-2 and -9 after 72 h treatment (Figure 1d).

### IL-1β induces a dose-dependent increase of the gelatinases secreted by mono-culture and co-culture chondrocytes

**Figure 2:**
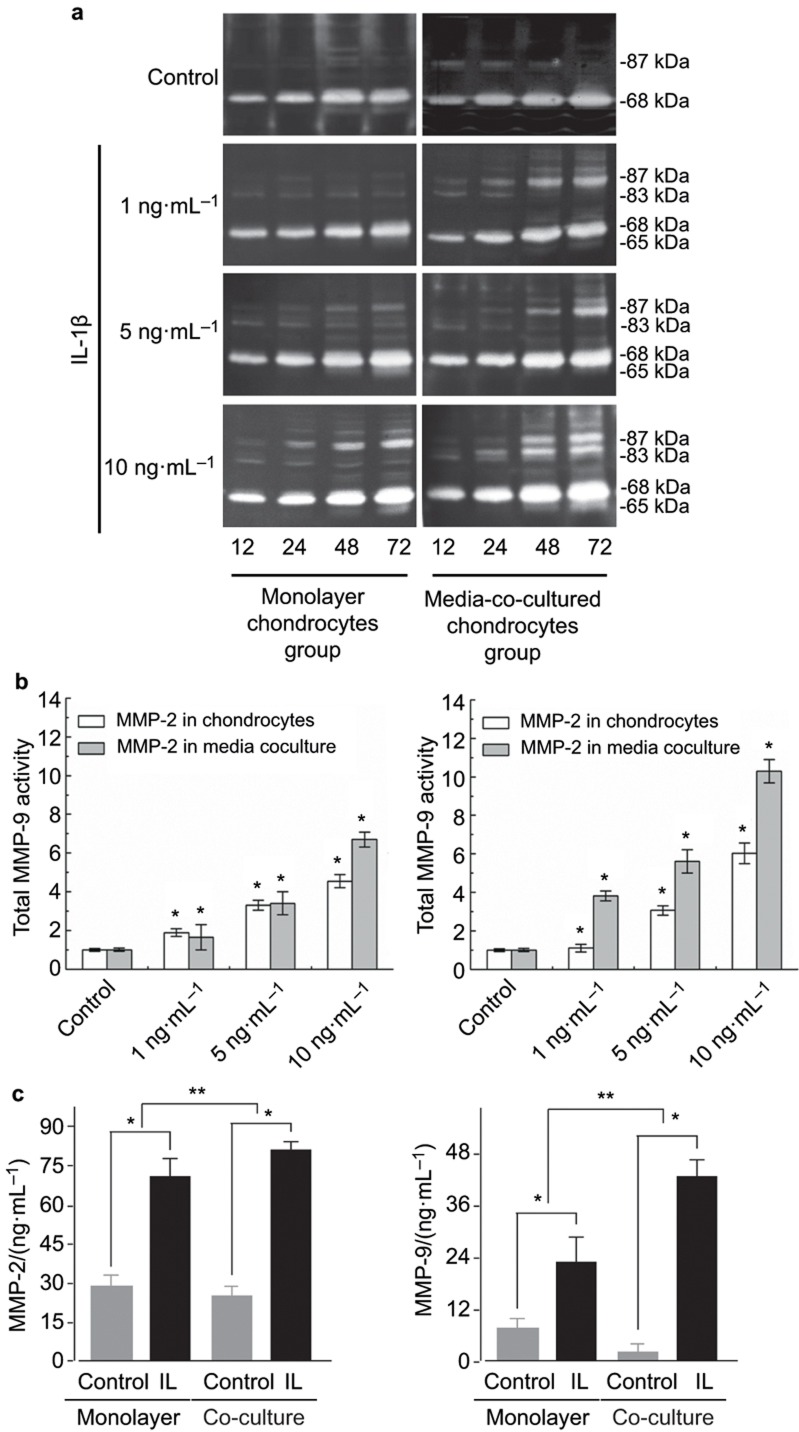
**IL-1β increases the activity of gelatinases in a dose-dependent manner in both mono-cultured and co-cultured chondrocytes.** (**a**) Zymography showed different dose-dependent increases of the gelatinases in mono-cultured and co-cultured chondrocytes. Pro-MMP-9 (87 kDa), active-MMP-9 (83 kDa), pro-MMP-2 (68 kDa) and active-MMP-2 (65 kDa) are in the right lane. The gels shown are representative of three different experiments (*n* = 3). (**b**) The quantification was performed with Quantity One 4.6.3 software. The optical densities of the pro- and active-MMP-2 and -9 bands were added as the total value of activity for MMP-2 and -9. The data are the mean of three different experiments (*n* = 3). *Significant difference with respect to control (*P* < 0.05). (**c**) ELISA Kit confirmed the IL-1β-induced gelatinases secreted by chondrocytes in mono-culture and co-culture groups (mean ± standard deviation) (*n* = 4). *P < 0.05, **P < 0.025, compared with controls. ELISA, enzyme linked immunosorbent assay; IL, interleukin; MMP, metalloproteinase.

Next, the activity of the gelatinases was examined after induction by exogenous IL-1β, which is commonly present *in vivo* in the micro-environment in the cartilage layer of inflamed OA. In the mono-culture group, IL-1β enhanced MMP-2 and -9 expressions in a time- and dose-dependent manner (Figure 2a). The total activity of MMP-2 and -9 increased 454% and 602%, respectively, after treatment with 10 ng·mL^−1^ (Figure 2b). In the co-culture group, the total MMP-2 activity was higher than in the mono-culture group (as high as 670% after treatment with 10 ng·mL^−1^ IL-1β, Figure 2b). Additionally, not only were the levels of pro-form of MMP-9 increased, but the active form was also prominent (as high as 1 030% after treatment with 10 ng·mL^−1^ IL-1β, Figure 2b), which was significantly different from the mono-cultured chondrocytes. We further examined the expressions of MMP-2 and -9 by ELISA and confirmed the IL-1β-induced MMP-2 and -9 in mono-cultured and co-cultured chondrocyte groups (Figure 2c).

### IL-1β restores the activity of gelatinases in MAPK inhibitor-pretreated chondrocytes, especially in osteoclast-conditioned media group

**Figure 3:**
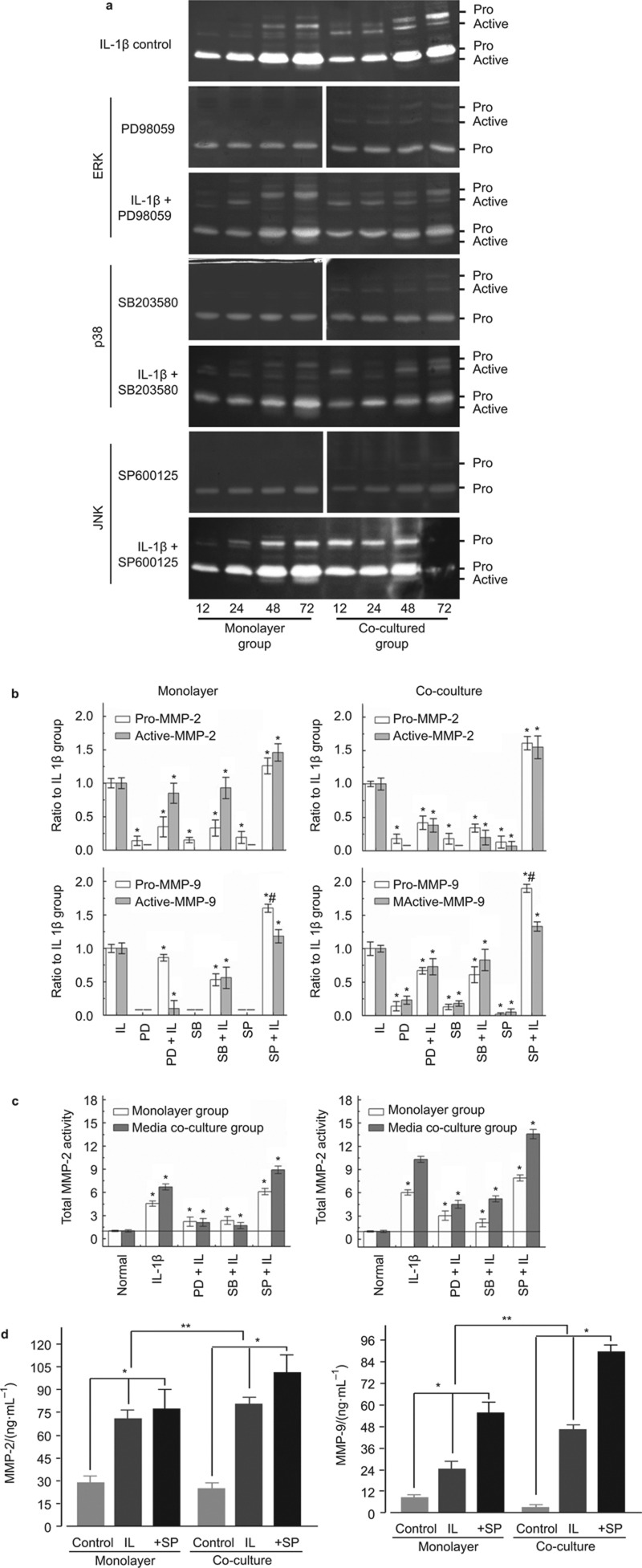
**IL-1β restores the high activities of gelatinases in MAPK-specific inhibitor-pretreated mono-cultured and co-cultured chondrocytes.** (**a**) Zymography demonstrating that IL-1β (10 ng·mL^−1^) can restore the activities of gelatinases through ERK (in the presence of PD58059), p38 (in the presence of SB203580) and especially JNK (in the presence of SP600125). The gels shown are representative of three different experiments (*n* = 3). (**b**) The activities of pro- and active-MMP-2/9 in mono-cultured and co-cultured chondrocytes were quantified by the optical densities methods (Quantity One 4.6.3 software). The indicated quantitative data refer to 72 h time-points. The data are the mean of three different experiments (*n* = 3). *Significant difference with respect to IL-1β-induced control (*P* < 0.05); ^#^Significant difference with respect to other two inhibitor-treated groups (*P* < 0.05). (**c**) IL-1β restored the total activities of gelatinases in MAPK inhibitor-pretreated chondrocytes (mean ± standard deviation) (*n* = 3). Scale bars, standard deviation. *P < 0.05, compared with control. (**d**) Due to high restored gelatinases in JNK inhibitor group, ELISA was done to explore the gelatinase secretion in both mono-culture and co-culture groups (mean ± standard deviation) (*n* = 4). *P < 0.05, **P < 0.025, compared with controls. ELISA, enzyme linked immunosorbent assay; ERK, extracellular signal-regulated kinase; IL, interleukin; JNK, c-Jun N-terminal kinase; MMP, metalloproteinase.

External antagonists have been shown to be effective on MMPs (e.g., MMP-1, -3 and -13) *in vitro*, but not *in vivo*.^10^ This issue can be studied by examining the intrinsic gelatinase modulation, which has been defined as the second step for collagen degradation (denatured collagen or gelatin). Here, we found that IL-1β treatment led to the retained activity of MMP-2 and -9 in specific MAPK inhibitor-pretreated chondrocytes in both mono-cultured and co-cultured chondrocytes. Inhibitor-pretreated chondrocytes had reduced gelatinase levels in all MAPK pathways, but the addition of IL-1β in inhibitor-pretreated chondrocytes restored the gelatinase activities (Figure 3a). Gelatinase restoration in the ERK and p38 pathways was weaker compared to IL-1β-induced control (ERK: The pro and active forms of MMP-2 were restored 35% and 85%, respectively, and the pro and active forms of MMP-9 were restored 86% and 10%, respectively; p38: the pro and active forms of MMP-2 were restored 33% and 93%, respctively, and the pro and active forms of MMP-9 were restored 53% and 56%, respectively, in the mono-culture group) (Figure 3b, left lane). In co-culture group, gelatinase restoration in ERK and p38 was lower in the co-culture group compared with the mono-culture group except for the active forms of MMP-9 in ERK and p38 (up to 4.87-fold and 1.48-fold, respectively, relative to those in the mono-culture group). In the JNK pathway, IL-1β not only totally restored MMP-2 and -9 expression, but also promoted higher expression in both mono-culture and co-culture groups (in mono-culture: 126% and 146% in the pro and active forms, respectively, of MMP-2; 160% and 118% in the pro and active forms, respectively, of MMP-9; in co-culture: 161% and 155% in the pro and active forms, respectively, of MMP-2; 195% and 133% in the pro and active forms, respectively, of MMP-9). We then calculated the total activities of the gelatinases among different IL-1β-treated groups. We found that IL-1β-restored gelatinase activity in ERK and p38 was generally lower compared with that in JNK, but still higher than that in normal chondrocytes in both the mono-culture and co-culture groups. In order to further confirm the IL-1β-induced gelatinases in the presence of JNK pathway inhibitor, we additionally used ELISA kit to explore the gelatinase expressions (Figure 3c).

We next examined the role of the NF-κB pathway by using Bay11-7082, the specific inhibitor of NF-κB p65 (Supplementary Figure S1). We found that Bay11-7082 significantly reduced the gelatinase expression. Furthermore, IL-1β could not restore gelatinase expression in Bay11-7082 pretreated mono-culture and co-culture chondrocytes, and the reduced gelatinases were present at similar levels to those in normal chondrocytes.

### IL-1β modulates the transcription of MMPs and TIMPs through MAPKs in mono-cultured and co-cultured chondrocytes

**Figure 4:**
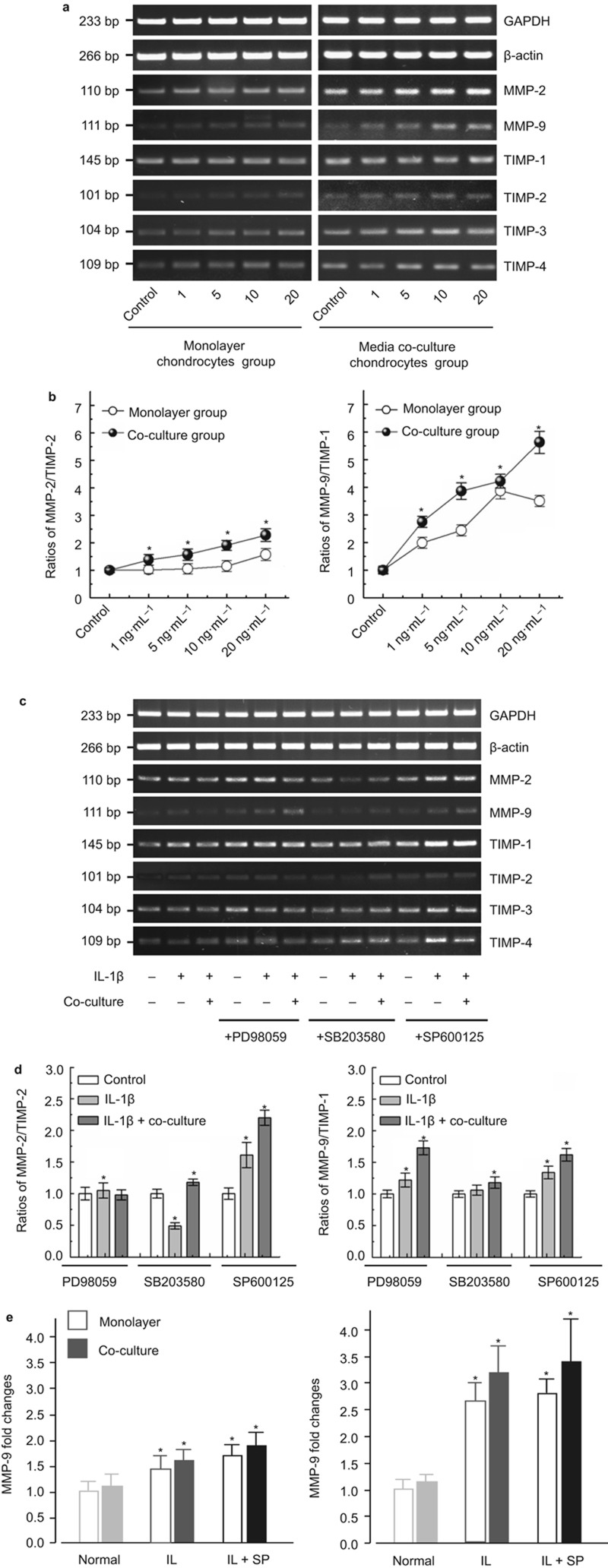
**IL-1β induced gene expression of gelatinases and TIMPs in MAPK-specific inhibitor-pretreated mono-cultured and co-cultured chondrocytes.** (**a**) IL-1β at different concentrations induces gene expression of gelatinases and TIMPs in mono-cultured and co-cultured chondrocytes.1, 5, 10 and 20 = 1, 5, 10 and 20 ng·mL^−1^. GAPDH and β-actin were used as the reference genes. The product sizes are indicated in the left lane. The gels shown are representative of three independent experiments (*n* = 3). (**b**) High ratios of MMP-2/TIMP-2 and MMP-9/TIMP-1 induced by different concentrations of IL-1β in inhibitor-pretreated mono-culture and co-culture chondrocytes. The data were the mean of three different experiments (*n* = 3). *Significant difference with respect to the normal ratios (*P* < 0.05). (**c**) The gene profiles of the gelatinases and TIMPs induced by IL-1β and/or specific inhibitors in mono-cultured and co-cultured chondrocytes. GAPDH and β-actin were used as internal controls. The product sizes are indicated in the left lane. The gels shown are representative of three independent experiments (*n* = 3). (**d**) Different ratios of MMP-2/TIMP-2 and MMP-9/TIMP-1 inhibitor-pretreated mono-cultured and media co-cultured chondrocytes. The data are the mean of three different experiments (*n* = 3). *Significant difference with respect to control ratios (*P* < 0.05). (**e**) qPCR was done to confirm the gene expressions of gelatinases induced by IL-1β in mono-culture and co-culture group. *Significant difference with respect to monolayer chondrocytes (*P* < 0.05). GAPDH, glyceraldehyde-3-phosphate dehydrogenase; IL, interleukin; MMP, metalloproteinase; TIMP, tissue inhibitors of metalloproteinase.

After the detection of post-transcriptional regulation of gelatinases, we examined the gene expression levels of IL-1β-induced gelatinases and TIMPs in specific inhibitor-pretreated mono-cultured and co-cultured chondrocytes (Figure 4). IL-1β increased the gene expression of MMP-2 and -9 in a dose-dependent manner in the both mono-cultured and co-cultured groups; TIMPs, especially TIMP-2, also increased in a dose-dependent manner (Figure 4a). Quantification by the optical density (OD) method showed higher ratios of MMP-2/TIMP-2 and MMP-9/TIMP-1 in the co-culture group compared with those in the mono-culture group (the former was as high as 2.28-fold, the latter was as high as 5.63-fold at 20 ng·mL^−1^) (Figure 4b).

Next, we found that IL-1β enhanced the gene expression of MMP-2 and -9 especially in SP600125 (JNK inhibitor)-pretreated mono-cultured and co-cultured chondrocytes. In the SB203580 (p38 MAPK inhibitor)-pretreated group, MMP-2 and -9 gene expression decreased, but in the PD98059 (ERK inhibitor)-pretreated group, MMP-9 was enhanced in the co-cultured chondrocytes (Figure 4c). Quantification by the OD method showed that the ratios of IL-1β-induced MMP-2/TIMP-2 in the SP600125-pretreated group were all higher compared to the other two pretreated groups (1.61-fold and 2.20-fold in mono-cultured and co-cultured chondrocytes, respectively, compared to the normal ratio, Figure 4d, left lane). The ratios of IL-1β-induced MMP-9/TIMP-1 in PD98059- and SP600125-pretreated chondrocytes were all higher than their normal ratios (ERK: 1.22-fold and 1.73-fold; JNK: 1.34-fold and 1.62-fold in mono-cultured and co-cultured chondrocytes, respectively, Figure 4d, right lane).

We then used qPCR to confirm the fold changes in the presence of JNK pathway inhibitor in which the gelatinase activity restored most. The results showed were in accordance with that of semi-quantitative PCR.

We also demonstrated that IL-1β enhanced both MMP-1 and -3 in the mono-cultured and co-cultured chondrocytes (Supplementary Figure S2).

## Discussion

By characterising the IL-1β-restored activities of the gelatinases between the inhibitor-pretreated mono-cultured and co-cultured chondrocytes, we provided novel insights into the influence of osteoclast signals on chondrocytes by indicating the conditioned medium derived from osteoclasts affected on chondrocytes. In this study, we found the following: (1) secreted factors from osteoclasts attenuated the activities of gelatinases; (2) IL-1β-induced activities of gelatinases in co-cultured chondrocytes were significantly higher than in mono-cultured chondrocytes; (3) IL-1β restored gelatinase activities in the MAPK inhibitor-pretreated chondrocytes; (4) IL-1β restored high gelatinase levels in co-cultured chondrocytes at the transcriptional level.

OA can be defined as a complex or interactive degradative and repair process involving the cartilage, subchondral bone and synovial membrane.^2,26^ Previous studies evaluated the expression levels of collagenases, cytokines and growth factors in experimental animal models of OA, which mimic the disease in humans.^27,28^ However, those studies were primarily restricted to examine molecular changes in the cartilage layer without integrating information from the surrounding synovial membrane or subchondral bone in response to the disease. In more recent studies, researchers have studied the crosstalk between articular cartilage and synovial membrane. D'Andrea P *et al.* found direct crosstalk between chondrocytes and synovial cells through enhanced intercellular calcium signalling.^4^ Dingle *et al.* found that factors released from synovium could enhance the catabolic metabolism of chondrocytes.^5^ In an *in vitro* model, the co-culture of injured cartilage with joint capsule explants enhanced the deleterious effects of injury on catabolic gene expression in cartilage and resulted in a reduction of cartilage aggrecan content.^29^ Macrophages from the inflamed synovium also showed direct communication with chondrocytes through proteases. 6 In our study, we found a crosstalk mechanism between chondrocytes and osteoclasts; this crosstalk modulated gelatinase activity.

Based on clinical evidence, cartilage damage is often accompanied by bone lesions.^6,30^*In vivo* data show that increased bone resorption occurs at an early stage in the development of OA and that blocking bone-resorbing cytokines prevents cartilage damage;^2,31^ these data infer the bone factors may play an important role in cartilage behaviour. We found that secreted factors from osteoclasts modulated the gelatinase activity in chondrocytes. Chondrocytes with osteoclast-conditioned medium secreted less gelatinase compared with mono-cultured chondrocytes *in vitro*. This may be a useful method to study the influence of factors from subchondral bone on cartilage.

The MMPs are a large group of enzymes that, due to their ability to degrade a wide variety of ECM components, play a crucial role in the destruction of cartilage and bone in an arthritic joint.^8^ The MMP family members produced by chondrocytes mainly consist of collagenases (MMP-1 and -13), stromelysin-1 (MMP-3) and gelatinases (MMP-2 and -9).^32^ MMP-1 is the most abundant member of the MMP family, and plays an important role in breaking cartilage-specific type II collagen.^10^ MMP-13 is another major enzyme that cleaves and denatures type II collagen in OA cartilage 5–10 times more effectively than MMP-1.^33^ MMP-3 can cleave proteoglycan and type IX collagen as well as type II collagen.^11^ As the degradation of type II collagen by MMP-2 and -9 is weaker (MMP-13 > MMP-1 > MMP-3 >> MMP-2 and -9),^34^ there are few reports that study its contribution to degradation. However, gelatinases effectively degrade denatured collagen (gelatins) caused by collagenases, which could induce further cartilage destruction.^12^ We found that IL-1β enhanced the activities of gelatinases in a dose-dependent manner in mono-cultured chondrocytes, which was in agreement with the activities of other MMPs in chondrocytes.^35^ In osteoclast-medium cultured chondrocytes, the enhanced activities of gelatinases by IL-1β were far higher, especially the active form of MMP-9, than those in mono-cultured chondrocytes.

For the past three decades, over 56 matrix metalloproteinase (MMP) inhibitors have been discovered as clinical candidates in various therapeutic areas including arthritis.^10^ Three of the specific inhibitors of MAPKs were used as therapeutic treatment for OA.^14,15^ Unfortunately, they failed to show benefit in clinical trials for various reasons including *in vivo* inefficacy and side effects such as arthralgia, myalgia and tendonitis.^36^ In the early stage of OA, the inhibition of p38 by SB203580 in bovine cartilage explants blocked IL-1-mediated collagen breakdown, while proteoglycan degradation was unaffected.^16^ PD98059 (an ERK inhibitor) can reduce IL-1β-induced MMP-1 mRNA expression in chondrocytes.^15^ An ERK pathway inhibitor (U0126), but not SB203580 (a p38-specific inhibitor) or SP600125 (a JNK-specific inhibitor), also selectively inhibited IL-1β-induced MMP-13 production in HAC.^37^ We found that IL-1β can restore the activity of gelatinases through MAPK inhibitors (especially SP600125), although this restoration can be eliminated by Bay 11-7082, a specific inhibitor of NF-κB. On the one hand, IL-1β-restored gelatinases that are restored through MAPKs could directly participate in chondrocyte damage through the degradation of ECM proteins (e.g., elastin, vitronectin and aggrecan) and non-ECM proteins (e.g., chemokines, myelin basic protein, amyloid beta peptide and substance P) in addition to denatured collagen (gelatin) and intact collagen type IV.^38^ On the other hand, highly expressed gelatinases activate other MMPs, especially MMP-1 and -13.^12^ This cascade recovers MMP networks, which shows the importance of gelatinases in ECM modulation in chondrocytes in the presence of the current therapeutic candidates.

The gelatinases differ from most other MMPs in that they have a collagen-binding domain (CBD) within the catalytic domain (Supplementary Figure S3). The CBD is composed of three fibronectin type II repeats and is involved in the binding of collagenous substrates, elastin, fatty acids and thrombospondins.^8^ MMP-2 expression is constitutive and most pro-inflammatory stimuli fail to increase its expression levels. In contrast to MMP-9, MMP-2 lacks binding sites for pro-inflammatory transcription factors such as activator protein-1.^25^ Meanwhile, MMP-2 knockout mice exhibit a normal phenotype under physiological conditions which indicate that MMP-2's function may be interchangeable with that of MMP-9.^39^ This hypothesis is supported by the observation that the expression of MMP-9 is greatly increased in MMP-2 null mice.^40^ We could infer that this interchangeable role of MMP-2 and -9 in the IL-1β-activated microenvironment might magnify both the collagen degradation and collagenase activation. This might be another piece of evidence supporting the role of gelatinases in recovering MMP networks and leading to the ECM imbalance in cartilage.

In addition to regulation at the protein level, the expression of gelatinases is also modulated at the transcriptional level via many factors such as the Raf/ERK pathway, MAPK/ERK/JNK pathway, Ras/MAPK pathway, NF-κB and activator protein-1.^41^ We examined the gene expression levels of gelatinases and their endogenous inhibitors, the TIMPs. In co-cultured chondrocytes, we found that IL-1β-regulated MMPs and TIMPs at the transcriptional level (Figure 4). As pro forms of MMP-2 and -9 are mainly inhibited by TIMP-2 and TIMP-1, respectively,^42^ we then examined the ratios of MMP-2/TIMP-2 and MMP-9/TIMP-1, which could indirectly reflect the transcriptional levels of gelatinases. IL-1β (10 ng·mL^−1^) induced high ratios (MMP-2/TIMP-2: 1.91-fold; MMP-9/TIMP-1: 4.22-fold). Moreover, IL-1β induced higher ratios in inhibitor-pretreated co-cultured chondrocytes through JNK (MMP-2/TIMP-2: 2.2-fold; MMP-9/TIMP-1: 1.48-fold compared with 10 ng·mL^−1^ IL-1β ratios). In addition, IL-1β also enhanced the expressions of other MMPs and TIMPs in inhibitor-pretreated mono-cultured and co-cultured chondrocytes (Figure 4c and Supplementary Figure S2).

There are limitations in this study. First, this is an *in vitro* model mimicking the chondrocyte microenvironment *in vivo* with secreted factors produced by osteoclasts. Other factors from bone tissue (osteoblasts and osteocyte) and factors from synovium and other joint tissues were not taken into account in this study, even though these factors together can modulate the networks of MMPs released from chondrocytes. This should be considered when we compare the MMPs data with the clinical results. Second, the chondrocytes are from 1-day-old mouse. Primary cultured chondrocytes might be a better model than chondrocyte cell lines such as Hum-Cell-0096 and HC-a 4650 (ScienCell, San Diego, CA, USA). However, this system does not exactly represent the chondrocytes as they exist in pathological condition. The further experiments using OA chondrocytes against various stimuli should be explored. Third, although this study elucidated the importance of gelatinases in chondrocytes with osteoclasts-conditioned medium *in vitro*, *ex vivo* and *in vivo* studies should be further confirmed.

## Figures and Tables

**Table 1:** Primers for housekeeping genes (GAPDH and β-actin), MMPs and TIMPs for semi-quantitative PCR

mRNA	Primer pairs
GAPDH (233 bp)	Forward: GGTGAAGGTCGGTGTGAACG
	Reverse: CTCGCTCCTGGAAGATGGTG
β-actin (266 bp)	Forward: GTCCCTCACCCTCCCAAAAG
	Reverse: GCTGCCTCAACACCTCAACCC
MMP-1 (106 bp)	Forward: TCATACTACCATCCTGCGACTC
	Reverse: TCACCTCTAAGCCAAAGAAAGA
MMP-2 (110 bp)	Forward: ATGTGTCTTCCCCTTCACTTTC
	Reverse: GGTCATCATCGTAGTTGGTTGT
MMP-3 (113 bp)	Forward: AAGGTCTGGGAGGAGGTGAC
	Reverse: CCATCAAAAGGGACAAAGTCTC
MMP-9 (111 bp)	Forward: CTTCCCCAAAGACCTGAAAAC
	Reverse: ACTGCTTCTCTCCCATCATCT
TIMP-1 (145 bp)	Forward: CTGGCATCCTCTTGTTGCTATC
	Reverse: AAGGTGGTCTCGTTGATTTCTG
TIMP-2 (101 bp)	Forward: TCTGAAGTCTGGTAGCCTGTGA
	Reverse: ACCGTTTCTTTGGGGTTTCT
TIMP-3 (104 bp)	Forward: CAGGGGAGTGTGAGTGTTAGGT
	Reverse: TGGGGAAGAAGTGTATGCTGTC
TIMP-4 (109 bp)	Forward: CTTGCGATGTGTGCTATGGTAG
	Reverse: TTGAGACAGTGGGAGTAGGAGAT

GAPDH, glyceraldehyde-3-phosphate dehydrogenase; MMP, metalloproteinase; PCR, polymerase chain reaction; TIMP, tissue inhibitors of metalloproteinase.

## References

[bib1] Wieland HA, Michaelis M, Kirschbaum BJ et al. Osteoarthritis – an untreatable disease? Nat Rev Drug Discov 2005; 4(4): 331–344. 15803196 10.1038/nrd1693

[bib2] Funck-Brentano T, Cohen-Solal M. Crosstalk between cartilage and bone: when bone cytokines matter. Cytokine Growth Factor Rev 2011; 22(2): 91–97. 21596615 10.1016/j.cytogfr.2011.04.003

[bib3] Haywood L, McWilliams DF, Pearson CI et al. Inflammation and angiogenesis in osteoarthritis. Arthritis Rheum 2003; 48(8): 2173–2177. 12905470 10.1002/art.11094

[bib4] D'andrea P, Calabrese A, Grandolfo M. Intercellular calcium signalling between chondrocytes and synovial cells in co-culture. Biochem J 1998; 329(Pt 3): 681–687. 9445399 10.1042/bj3290681 PMC1219093

[bib5] Dingle JT, Saklatvala J, Hembry R et al. A cartilage catabolic factor from synovium. Biochem J 1979; 184(1): 177–180. 534517 10.1042/bj1840177 PMC1161690

[bib6] Dreier R, Wallace S, Fuchs S et al. Paracrine interactions of chondrocytes and macrophages in cartilage degradation: articular chondrocytes provide factors that activate macrophage-derived pro-gelatinase B (pro-MMP-9). J Cell Sci 2001; 114(Pt 21): 3813–3822. 11719548 10.1242/jcs.114.21.3813

[bib7] Sandy JD. A contentious issue finds some clarity: on the independent and complementary roles of aggrecanase activity and MMP activity in human joint aggrecanolysis. Osteoarthr Cartil 2006; 14(2): 95–100. 16257242 10.1016/j.joca.2005.09.004

[bib8] Page-McCaw A, Ewald AJ, Werb Z. Matrix metalloproteinases and the regulation of tissue remodelling. Nat Rev Mol Cell Biol 2007; 8(3): 221–233. 17318226 10.1038/nrm2125 PMC2760082

[bib9] Elliott SF, Coon CI, Hays E et al. Bcl-3 is an interleukin-1-responsive gene in chondrocytes and synovial fibroblasts that activates transcription of the matrix metalloproteinase 1 gene. Arthritis Rheum 2002; 46(12): 3230–3239. 12483727 10.1002/art.10675

[bib10] Takaishi H, Kimura T, Dalal S et al. Joint diseases and matrix metalloproteinases: a role for MMP-13. Curr Pharm Biotechnol 2008; 9(1): 47–54. 18289056 10.2174/138920108783497659

[bib11] Grottkau BE, Lin Y. Osteogenesis of adipose-derived stem cells. Bone Res 2013; 1(2): 133–145. 26273498 10.4248/BR201302003 PMC4472098

[bib12] Sang QA, Bodden MK, Windsor LJ. Activation of human progelatinase A by collagenase and matrilysin: activation of procollagenase by matrilysin. J Protein Chem 1996; 15(3): 243–253. 8804571 10.1007/BF01887112

[bib13] Geng Y, Valbracht J, Lotz M. Selective activation of the mitogen-activated protein kinase subgroups c-Jun NH2 terminal kinase and p38 by IL-1 and TNF in human articular chondrocytes. J Clin Invest 1996; 98(10): 2425–2430. 8941662 10.1172/JCI119056 PMC507695

[bib14] Johnson GL, Lapadat R. Mitogen-activated protein kinase pathways mediated by ERK, JNK, and p38 protein kinases. Science 2002; 298(5600): 1911–1912. 12471242 10.1126/science.1072682

[bib15] Raymond L, Eck S, Mollmark J et al. Interleukin-1 beta induction of matrix metalloproteinase-1 transcription in chondrocytes requires ERK-dependent activation of CCAAT enhancer-binding protein-beta. J Cell Physiol 2006; 207(3): 683–688. 16453302 10.1002/jcp.20608

[bib16] Mengshol JA, Vincenti MP, Coon CI et al. Interleukin-1 induction of collagenase 3 (matrix metalloproteinase 13) gene expression in chondrocytes requires p38, c-Jun N-terminal kinase, and nuclear factor kappaB: differential regulation of collagenase 1 and collagenase 3. Arthritis Rheum 2000; 43(4): 801–811. 10765924 10.1002/1529-0131(200004)43:4<801::AID-ANR10>3.0.CO;2-4

[bib17] Fruebis J, Tsao TS, Javorschi S et al. Proteolytic cleavage product of 30-kDa adipocyte complement-related protein increases fatty acid oxidation in muscle and causes weight loss in mice. Proc Natl Acad Sci U S A 2001; 98(4): 2005–2010. 11172066 10.1073/pnas.041591798 PMC29372

[bib18] Long DL, Loeser RF. p38gamma mitogen-activated protein kinase suppresses chondrocyte production of MMP-13 in response to catabolic stimulation. Osteoarthr Cartil 2010; 18(9): 1203–1210. 20633667 10.1016/j.joca.2010.05.016 PMC2929282

[bib19] Alge-Priglinger CS, Kreutzer T, Obholzer K et al. Oxidative stress-mediated induction of MMP-1 and MMP-3 in human RPE cells. Invest Ophthalmol Vis Sci 2009; 50(11): 5495–5503. 19516002 10.1167/iovs.08-3193

[bib20] Nakai K, Kawato T, Morita T et al. Angiotensin II induces the production of MMP-3 and MMP-13 through the MAPK signaling pathways via the AT_1_ receptor in osteoblasts. Biochimie 2013; 95(4): 922–933. 23277113 10.1016/j.biochi.2012.12.016

[bib21] Vu TH, Shipley JM, Bergers G et al. MMP-9/gelatinase B is a key regulator of growth plate angiogenesis and apoptosis of hypertrophic chondrocytes. Cell 1998; 93(3): 411–422. 9590175 10.1016/s0092-8674(00)81169-1 PMC2839071

[bib22] Crane JL, Zhao L, Frye JS et al. IGF-1 signaling is essential for differentiation of mesenchymal stem cells for peak bone mass. Bone Res 2013; 1(2): 186–194. 26273502 10.4248/BR201302007 PMC4472101

[bib23] Kadri A, Funck-Brentano T, Lin H et al. Inhibition of bone resorption blunts osteoarthritis in mice with high bone remodelling. Ann Rheum Dis 2010; 69(8): 1533–1538. 20525838 10.1136/ard.2009.124586

[bib24] Xie J, Wang C, Huang DY et al. TGF-beta1 induces the different expressions of lysyl oxidases and matrix metalloproteinases in anterior cruciate ligament and medial collateral ligament fibroblasts after mechanical injury. J Biomech 2013; 46(5): 890–898. 23357697 10.1016/j.jbiomech.2012.12.019

[bib25] Pickarski M, Hayami T, Zhuo Y et al. Molecular changes in articular cartilage and subchondral bone in the rat anterior cruciate ligament transection and meniscectomized models of osteoarthritis. BMC Musculoskelet Disord 2011; 12: 197. 21864409 10.1186/1471-2474-12-197 PMC3176489

[bib26] Aigner T, Zien A, Gehrsitz A et al. Anabolic and catabolic gene expression pattern analysis in normal versus osteoarthritic cartilage using complementary DNA-array technology. Arthritis Rheum 2001; 44(12): 2777–2789. 11762938 10.1002/1529-0131(200112)44:12<2777::aid-art465>3.0.co;2-h

[bib27] Daouti S, Latario B, Nagulapalli S et al. Development of comprehensive functional genomic screens to identify novel mediators of osteoarthritis. Osteoarthr Cartil 2005; 13(6): 508–518. 15922185 10.1016/j.joca.2005.02.003

[bib28] Lee JH, Fitzgerald JB, DiMicco MA et al. Co-culture of mechanically injured cartilage with joint capsule tissue alters chondrocyte expression patterns and increases ADAMTS5 production. Arch Biochem Biophys 2009; 489(1/2): 118–126. 19607802 10.1016/j.abb.2009.07.006 PMC2752630

[bib29] Radin EL, Rose RM. Role of subchondral bone in the initiation and progression of cartilage damage. Clin Orthop Relat Res 1986; (213): 34–40. 3780104

[bib30] Radin EL, Paul IL, Tolkoff MJ. Subchondral bone changes in patients with early degenerative joint disease. Arthritis Rheum 1970; 13(4): 400–405. 4246869 10.1002/art.1780130406

[bib31] Grottkau BE, Purudappa PP, Lin YF. Multilineage differentiation of dental pulp stem cells from green fluorescent protein transgenic mice. Int J Oral Sci 2010; 2(1): 21–27. 20690415 10.4248/IJOS10015 PMC3475594

[bib32] Mitchell PG, Magna HA, Reeves LM et al. Cloning, expression, and type II collagenolytic activity of matrix metalloproteinase-13 from human osteoarthritic cartilage. J Clin Invest 1996; 97(3): 761–768. 8609233 10.1172/JCI118475 PMC507114

[bib33] El Mabrouk M, Sylvester J, Zafarullah M. Signaling pathways implicated in oncostatin M-induced aggrecanase-1 and matrix metalloproteinase-13 expression in human articular chondrocytes. Biochim Biophys Acta 2007; 1773(3): 309–320. 17208315 10.1016/j.bbamcr.2006.11.018

[bib34] Itthiarbha A, Phitak T, Sanyacharernkul S et al. Polyoxypregnane glycoside from *Dregea volubilis* extract inhibits IL-1β-induced expression of matrix metalloproteinase via activation of NF-κB in human chondrocytes. In Vitro Cell Dev Biol Anim 2012; 48(1): 43–53. 22179678 10.1007/s11626-011-9475-7

[bib35] Rizvi NA, Humphrey JS, Ness EA et al. A phase I study of oral BMS-275291, a novel nonhydroxamate sheddase-sparing matrix metalloproteinase inhibitor, in patients with advanced or metastatic cancer. Clin Cancer Res 2004; 10(6): 1963–1970. 15041713 10.1158/1078-0432.ccr-1183-02

[bib36] Kimura H, Yukitake H, Suzuki H et al. The chondroprotective agent ITZ-1 inhibits interleukin-1 beta-induced matrix metalloproteinase-13 production and suppresses nitric oxide-induced chondrocyte death. J Pharmacol Sci 2009; 110(2): 201–211. 19542681 10.1254/jphs.09076fp

[bib37] Prasadam I, Farnaghi S, Feng JQ et al. Impact of extracellular matrix derived from osteoarthritis subchondral bone osteoblasts on osteocytes: role of integrin β1 and focal adhesion kinase signaling cues. Arthritis Res Ther 2013; 15(5): R150. 24289792 10.1186/ar4333 PMC3978998

[bib38] Corry DB, Rishi K, Kanellis J et al. Decreased allergic lung inflammatory cell egression and increased susceptibility to asphyxiation in MMP2-deficiency. Nat Immunol 2002; 3(4): 347–353. 11887181 10.1038/ni773 PMC2814346

[bib39] Esparza J, Kruse M, Lee J et al. MMP-2 null mice exhibit an early onset and severe experimental autoimmune encephalomyelitis due to an increase in MMP-9 expression and activity. FASEB J 2004; 18(14): 1682–1691. 15522913 10.1096/fj.04-2445com

[bib40] Zhang D, Bar-Eli M, Meloche S et al. Dual regulation of MMP-2 expression by the type 1 insulin-like growth factor receptor: the phosphatidylinositol 3-kinase/Akt and Raf/ERK pathways transmit opposing signals. J Biol Chem 2004; 279(19): 19683–19690. 14993222 10.1074/jbc.M313145200

[bib41] Al-Hizab F, Clegg PD, Thompson CC et al. Microscopic localization of active gelatinases in equine osteochondritis dissecans (OCD) cartilage. Osteoarthr Cartil 2002; 10(8): 653–661. 12479388 10.1053/joca.2002.0811

[bib42] Li P, Hu M, Sun S et al. Fluid flow-induced calcium response in early or late differentiated osteoclasts. Ann Biomed Eng 2012; 40(9): 1874–1883. 22532320 10.1007/s10439-012-0554-z

