# Supplementary material for: The effects of interleukin-1β in modulating osteoclast-conditioned medium's influence on gelatinases in chondrocytes through mitogen-activated protein kinases
Source: Int J Oral Sci. 2015 Oct 30;7(4):220–31. doi: 10.1038/ijos.2015.39 (PMC5153595; doi:10.1038/ijos.2015.39)

**The effects of IL-1β in modulating osteoclast-conditioned medium's influence on gelatinases in chondrocytes through MAP kinases**

Jing Xie^1^, Na Fu^1^, Linyi Cai^1^, Tao Gong^1^, Guo Li^1^, Qiang Peng^1^, Xiaoxiao Cai^1*^

^1^State Key Laboratory of Oral Diseases, West China Hospital of Stomatology, Sichuan University, Chengdu, Sichuan Province, P.R. China

Supplementary tables and figures

Table 1. **Primers for house keeping genes (GAPDH & β-actin), matrix metalloproteases (MMPs) and tissue inhibitors of matrix metalloproteases (TIMPs) for semi-quantitative PCR.**

Figure S1 (This figure was related to figure 3)

**IL-1β-induced activities of gelatinases could be reduced in NF-κB (p65) inhibitor-pretreated mono-culture and co-culture chondrocytes.** (A) Zymography showed the IL-1β-induced activities of gelatinases in inhibitor-pretreated mono-culture and co-culture chondrocytes. The gels shown were representatives of three different experiments (n = 3). (B) Quantitative analysis of zymography with Bio-Rad Image software (Quantity One 4.6.3 software). The data were the mean of three different experiments (n = 3); * Significant difference with respect to IL-1β control (*p* < 0.05).

Figure S2 (This figure was related to figure 4)

**IL-1β-induced gene expressions of MMP-1 and -3 in NF-κB (p65) inhibitor-pretreated mono-culture and co-culture chondrocytes.** GAPDH and β-actin were used as internal controls. The product sizes were indicated in the left lane. The gels shown were representative of three independent experiments (n = 3).

Figure S3

**Schematic diagram elucidated the structure of gelatinases.**

Table 1

| mRNA | Primer pairs |
| --- | --- |
| GAPDH (233bp)  β-ACTIN (266bp)  MMP-1(106bp)  MMP-2 (110bp)  MMP-3(113bp)  MMP-9 (111bp)  TIMP-1 (145bp)  TIMP-2 (101bp)  TIMP-3 (104bp)  TIMP-4 (109bp) | Forward GGTGAAGGTCGGTGTGAACG  Reverse CTCGCTCCTGGAAGATGGTG  Forward GTCCCTCACCCTCCCAAAAG  Reverse GCTGCCTCAACACCTCAACCC  Forward TCATACTACCATCCTGCGACTC  Reverse TCACCTCTAAGCCAAAGAAAGA  Forward ATGTGTCTTCCCCTTCACTTTC  Reverse GGTCATCATCGTAGTTGGTTGT  Forward AAGGTCTGGGAGGAGGTGAC  Reverse CCATCAAAAGGGACAAAGTCTC  Forward CTTCCCCAAAGACCTGAAAAC  Reverse GACTGCTTCTCTCCCATCATCT  Forward CTGGCATCCTCTTGTTGCTATC  Reverse AAGGTGGTCTCGTTGATTTCTG  Forward TCTGAAGTCTGGTAGCCTGTGA  Reverse ACCGTTTCTTTGGGGTTTCT  Forward CAGGGGAGTGTGAGTGTTAGGT  Reverse TGGGGAAGAAGTGTATGCTGTC  Forward CTTGCGATGTGTGCTATGGTAG  Reverse TTGAGACAGTGGGAGTAGGAGAT |

Figure S1


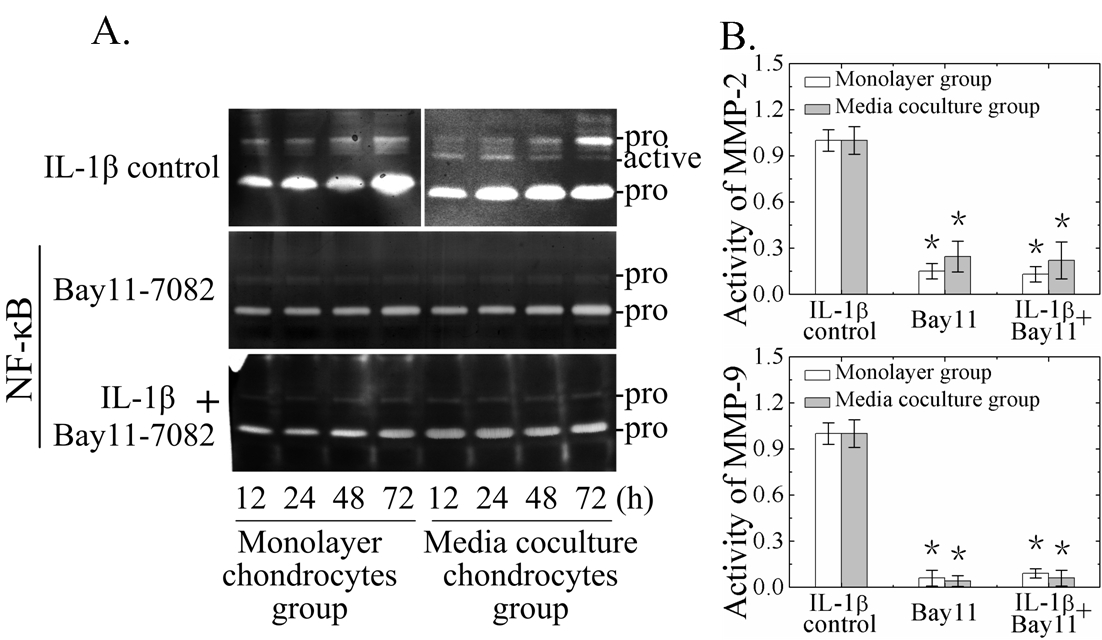


Figure S2


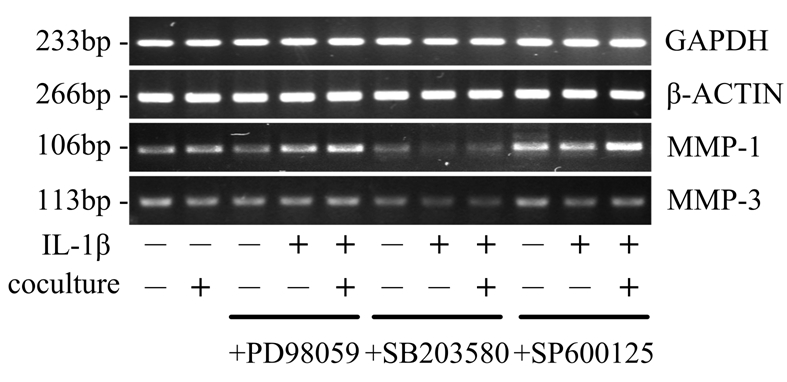


Figure S3


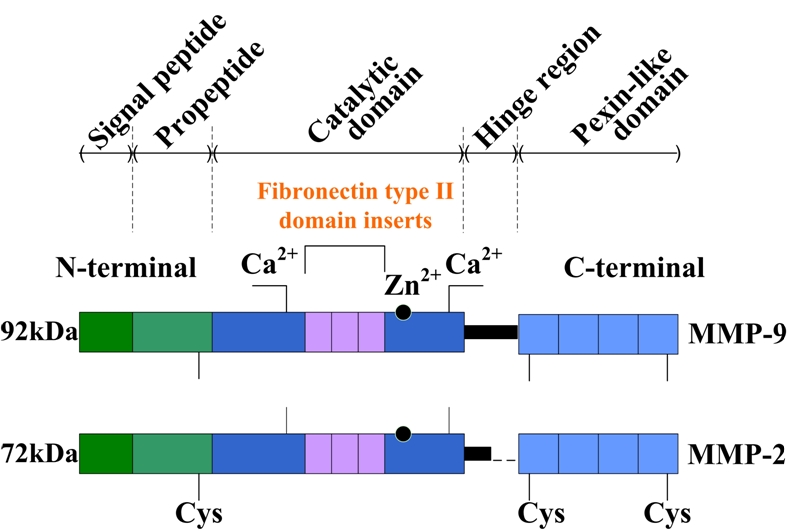

Supplement: Supplementary information [file ijos201539x1.doc]
